# The role of maternal ideations on breastfeeding practices in northwestern Nigeria: a cross-section study

**DOI:** 10.1186/s13006-022-00500-w

**Published:** 2022-09-01

**Authors:** Udochisom C. Anaba, Emily White Johansson, Dele Abegunde, Gloria Adoyi, Olayinka Umar-Farouk, Shittu Abdu-Aguye, Paul C. Hewett, Paul L. Hutchinson

**Affiliations:** 1 Breakthrough RESEARCH/Nigeria, Plot 839 Idris Ibrahim Crescent, Jabi, Abuja, Nigeria; 2 Department of Global Community Health and Behavioral Sciences, School of Public Health and Tropical Medicine, Tulane University, New Orleans, USA; 3 Formerly Population Council, Washington DC, USA; 4 Breakthrough ACTION/Nigeria, Abuja, Nigeria; 5 Save the Children, Abuja, Nigeria; 6 Johns Hopkins Center for Communication Programs, Baltimore, MD USA

**Keywords:** Exclusive breastfeeding, Early initiation of breastfeeding, Ideations, Psychosocial factors, Maternal health, Child health, Nutrition

## Abstract

**Background:**

Early initiation of breastfeeding within the first hour of birth and exclusive breastfeeding (EBF) for the first six months of life are beneficial for child survival and long-term health. Yet breastfeeding rates remain sub-optimal in Northwestern Nigeria, and such practices are often influenced by complex psychosocial factors at cognitive, social and emotional levels. To understand these influences, we developed a set of breastfeeding-related ideational factors and quantitatively examined their relationship with early initiation of breastfeeding and EBF practices.

**Methods:**

A cross‐sectional population‐based survey was conducted in Kebbi, Sokoto, and Zamfara states from September–October 2019. A random sample of 3039 women with a child under-2 years was obtained. Respondents were asked about the two main outcomes, early initiation of breastfeeding and EBF, as well as breastfeeding-related ideations according to the Ideation Model of Strategic Communication and Behavior Change. Average marginal effects were estimated from mixed-effects logistic regression models adjusted for ideational and socio-demographic variables.

**Results:**

Among 3039 women with a child under 2 years of age, 42.1% (95% CI 35.1%, 49.4%) practiced early initiation of breastfeeding, while 37.5% (95% CI 29.8%, 46.0%) out of 721 infants aged 0–5 months were exclusively breastfed. Women who knew early initiation of breastfeeding was protective of newborn health had 7.9 percentage points (pp) [95% CI 3.9, 11.9] higher likelihood of early initiation of breastfeeding practice than those who did not know. Women who believed colostrum was harmful had 8.4 pp lower likelihood of early initiation of breastfeeding (95% CI -12.4, -4.3) and EBF (95% CI -15.7%, -1.0%) than those without that belief. We found higher likelihood of early initiation of breastfeeding (5.1 pp, 95% CI 0.8%, 9.4%) and EBF (13.3 pp, 95% CI 5.0%, 22.0%) among women who knew at least one benefit of breastfeeding compared to those who did not know. Knowing the timing for introducing complementary foods andself-efficacy to practice EBF were also significantly associated with EBF practices.

**Conclusion:**

Ideational metrics provide significant insights for SBC programs aiming to change and improve health behaviors, including breastfeeding practices, Various cognitive, emotional and social domains played a significant role in women’s breastfeeding decisions. Maternal knowledge about the benefits of breastfeeding to the mother (cognitive), knowledge of the appropriate time to introduce complementary foods (cognitive), beliefs on colostrum (cognitive), self-efficacy to breastfeed (emotional) and perceived social norms (social) are among the most important ideations for SBC programs to target to increase early initiation of breastfeeding and EBF rates in northwestern Nigeria.

## Background

Breastfeeding remains an important cornerstone for child survival and long-term health because it provides essential nutrition and protection crucial for early growth and development [1]. Globally, optimal breastfeeding can avert more than 800,000 child deaths annually, which makes it one of the most effective child mortality prevention interventions with long-term health benefits [2]. Due to these beneficial effects of breastmilk, the World Health Organization (WHO) and the United Nations Children’s Fund (UNICEF) recommend early initiation of breastfeeding within one hour of birth, exclusive breastfeeding (EBF) for the first six months of life and continuous breastfeeding for up to two years of age with adequate complementary feeding [3]. These three recommendations constitute the indicators for optimal breastfeeding and nutrition in children.

The ‘Global Breastfeeding Collective’ set a global target rate of 70% for both early initiation of breastfeeding within one hour of birth and EBF for the first six months of life by 2030 as a requirement to protect the health of mothers and infants [4]. Despite the evidence of the beneficial effects of breastfeeding, optimal breastfeeding practices remain low in Nigeria. According to the 2018 Nigeria Demographic and Health Survey (NDHS) [5], national early initiation of breastfeeding and EBF rates were only 32% and 29% respectively. The northwestern region has some of the lowest rates for these practices in the country with only 32% of children breastfed within one hour of birth and 19% exclusively breastfed for the first six months of life [5]. In addition, the northwestern region also has the highest number of stunted children, highest under-5 mortality rate and lowest percentage of deliveries in health facilities [5].

Recent studies have explored factors that may influence early initiation of breastfeeding and EBF practices in northwestern Nigeria [6, 7]. Socio-demographic factors that were found to be associated with breastfeeding practices included maternal education, maternal employment, household wealth, antenatal care attendance and facility delivery [8, 9]. Previous qualitative research [10] conducted among women in northwestern Nigeria suggests that colostrum is perceived as not pure and potentially harmful to the newborn*,* prompting the common practice of introducing food and liquid early. In another qualitative study, authors [11] found that early introduction of foods and liquids may also stem from women’s perceptions of breast milk as insufficient, beliefs around infant’s thirst and need for water, as well as exclusive breastfeeding not being culturally acceptable including family attitudes such as husband’s disapproval. Elsewhere, studies [12–14] have revealed that community and family members’ cultural values and beliefs towards breastfeeding may influence a mother’s decision to breastfeed. Additional factors were also linked to breastfeeding behaviors, including knowledge, norms, self-efficacy, and other psychosocial influences [15, 16].

There has been recent and growing interest in developing metrics that quantitatively capture psychosocial influences, or ideations, that are viewed as intermediate determinants of behaviors [17, 18]. Ideation can be defined as “how new ways of thinking (or new behaviors) are diffused through a community by means of communication and social interaction among individuals and groups” [19]. These ideations are a primary focus of social and behavior change (SBC) programs [15, 20] as an intermediate pathway to positive health behaviors. Our study builds on the Ideation Model of Strategic Communication and Behavior Change [15, 21] that links individual ideation with behavior and incorporates constructs from cognitive, emotional and social domains derivative from several behavioral theories and models [22–25].

Existing behavioral theories emphasize different factors and variables associated with behavior change. For example, some emphasize the psychosocial concepts underlying the cognitive domain which includes knowledge, beliefs, values and attitudes [22]; others emphasize the social domains such as social influence, social support, spousal communication, and personal advocacy [25], while others focus on the emotional domain which includes fear, empathy and confidence or self-efficacy [26]. Emphasis on social and psychological determinants of behavior has been the most widely used strategy in many Social and Behavior Change Communication (SBCC) programs, but the Ideation model is unique because, firstly, it emphasizes the complexity of an individual’s decision-making process leading to behavior change, which usually involves multiple behavior-specific ideational variables acting simultaneously[27]. Secondly, these multiple behavior-specific ideational variables exert a cumulative influence, that is, the probability of behavior change is higher when more variables are positive in relation to the behavior [27]. Lastly, these ideations can be reinforced through social interaction and communication forms like exposure to mass media [28], which increases the probability of behavior change at the population level.

Ideations, which have been theorized as key variables that influence behavior [29] have been examined across health areas such as malaria [27, 30, 31] family planning [16, 32], routine vaccination [33] and pneumonia care-seeking and treatment [34]. In this paper, we examined the relationship between women’s breastfeeding behaviors and ideations (e.g., knowledge, beliefs, norms) in the northwestern Nigeria context to understand the most important ideations that could influence early initiation of breastfeeding and exclusive breastfeeding practices as a means to inform SBC programs in this region.

## Methods

### Study settings

This study was conducted in Kebbi, Sokoto, and Zamfara states in northwestern Nigeria within wards targeted for a USAID‐funded health‐related SBC program. One of the aims of the ongoing SBC program is to collect and report on a selection of ideational and priority behavioral indicators across malaria, family planning, maternal, newborn, and child health and nutrition (MNCH + N) in Sokoto, Kebbi and Zamfara States. As part of an evaluation of program impact, a baseline survey was carried out before program implementation in September 2019, of which ideational indicators for breastfeeding and other priority behaviors were collected.

### Study design

A two‐stage cluster‐sample cross‐sectional population‐based survey of women 15–49 years with a child under the age of two years living in wards within Kebbi, Sokoto, and Zamfara States targeted for health‐related SBC programming was conducted. The survey sample size was based on an evaluation design with three comparison groups. Sample size estimation allowed for a 10% non-response rate, a power criterion of 0.80, an alpha coefficient of 0.05, and varying intra-cluster correlations and minimal detectable differences for priority outcomes of the evaluation across the study arms. A sample size of 3039 women with a child under 2 years was targeted at baseline.

For the baseline survey, the first sampling stage included 108 enumeration areas (EAs) from SBC program wards within the three states (36 EAs per state), selected using digital mapping and a grid sampling methodology. Within sampled EAs, all households were enumerated, and women with a child under two years of age were randomly selected. In each sampled household, an eligible woman was asked to respond to the interview questionnaire.

### Data source

We collected data for this study as part of a baseline survey for the evaluation of an ongoing SBC program. There were two questionnaires (household and female questionnaires), the household questionnaire collected information on usual resident household members, bed net ownership and use, and household assets and characteristics. The female questionnaire asked all respondents about their demographics, reproductive history, contraceptive use, media exposure, gender norms and ideations related to family planning, malaria, and MNCH + N. Face-to-face, enumerator-directed interviews were used to obtain these data. All women aged 15 – 49 years with a child under two years of age were asked about their behaviors referenced to their last‐born child within the past two years. Behaviors included antenatal and delivery care, newborn care, routine vaccination, malaria prevention and treatment, child and maternal nutrition, childhood illness care‐seeking and treatment, and breastfeeding.

Training of supervisors and fieldwork staff occurred over one week in September 2019, and covered the study objectives, survey instrument reviews, ethical considerations, fieldwork procedures and participation in a questionnaire pilot exercise. Data were collected over four weeks in September 2019 through October 2019. Questionnaires were translated into Hausa (the predominat language), and pre‐tested to confirm translations, skip patterns, question appropriateness and sequencing. Questionnaires were similar in format and wording to the Demographic and Health Surveys (DHS), capturing behavioral outcomes. The ideational questions developed and measured for the survey and used in this analysis are described in Table 1.

**Table 1:** Breastfeeding-related ideational variable measurement

Ideational Dimension	Domain	Question or Likert Scale	Response categories
Cognitive	Knowledge	In your opinion, what is the ideal age to begin introducing complementary food in addition to breastmilk?	Spontaneously Reports 6 + months vs other responses
		In your opinion, what are the benefits, if any, for mothers who exclusively breastfeed their infant for the first six months of life?	Spontaneously reports any benefits vs no benefits or don’t know
		What can a mother do to protect the health of her newborn baby immediately after delivery?	Spontaneously mentions breastfeeding immediately within one-hour vs other responses or don’t know
	Beliefs	Breastmilk contains all the nutrients a baby needs during the first 6 months of his/her life	Agreed (strongly or somewhat) versus disagreed (strongly or somewhat) or don’t know
		A mother's breastmilk immediately after birth is bad milk	Agreed (strongly or somewhat) versus disagreed (strongly or somewhat) or don’t know
Emotional	Self-efficacy	How confident are you that you could exclusively breastfeed your child for the first six months of life?	Confident (very or somewhat) versus uncertain (very or somewhat) or don’t know
		How confident are you that you could start a conversation with your husband/partner about breastfeeding your child?	Confident (very or somewhat) versus uncertain (very or somewhat) or don’t know
Social	Social influence	Besides yourself, who else may influence your decision about whether to breastfeed or not?	Spontaneously reports no one else or spouse or mother-in-law or health provider
	Injunctive norms	It is important for mothers to only give their child breastmilk during the first 6 months after birth	Agreed (strongly or somewhat) versus disagreed (strongly or somewhat) or don’t know
	Descriptive norms	Most women in my community only give their infants breastmilk, and no water, for the first six months after birth	Agreed (strongly or somewhat) versus disagreed (strongly or somewhat) or don’t know

### Outcome variables

Two outcome variables were captured in this study: early initiation of breastfeeding within one hour of birth and exclusive breastfeeding among infants aged 0–5 months of age. For early initiation of breastfeeding, the woman was asked during the survey interview how long after birth she first put her child to the breast, and early initiation of breastfeeding outcome was measured as infants who initiated breastfeeding within 1 h of birth. We categorized our binary outcome for early initiation of breastfeeding as ‘1’ if a woman initiated breastfeeding within the first hour after birth and ‘0’ if otherwise.

For EBF, we calculated this as the proportion of last-born infants aged 0 – 5 months who were exclusively fed with breastmilk. Currently breastfeeding women were asked two questions for measuring EBF using the 24-h recall and first three days after birth definitions (1) If any soft or semi-solid food in addition to breastmilk has been given to the child in the past 24 h. (2) If any fluids or food items other than breastmilk had been given to the child in the first three days after birth. EBF outcome variable was measured as infants aged 0–5 months, currently breastfeeding without any liquids given to him/her during the first three days after birth and without any soft or semi-solid foods given to him/her in the previous 24 h of the survey. For EBF, we categorized our binary outcome as ‘1’ if a woman practiced EBF and ‘0’ if otherwise. This definition of exclusive breastfeeding slightly differs from the one reported in Demographic and Health Surveys. The Nigeria Demographic and Health Survey (NDHS) defined exclusive breastfeeding as the “proportion of children aged 0–5 months who are fed exclusively with breastmilk in the first 6 months of life”. Our definition of exclusive breastfeeding was harmonized for the NDHS.

Table 1 presents breastfeeding‐related ideations which are the main explanatory variables in this analysis. These ideations were developed based on the Ideational Model of Strategic Communication and Behavior Change [15], adapted as appropriate from previous research in other health areas such as malaria, family planning and vaccination [16, 27, 30–33]. A 5-point Likert response item was dichotomized to one category that collapsed “Strongly Disagree/Very Uncertain”, “Somewhat Disagree/Somewhat Uncertain, “Don’t know”, and another category for “Somewhat Agree/Somewhat Confident”, “Strongly Agree/Very Confident. This grouping choice was done to level the distribution of the original categories and to improve the intelligibility of our data [35, 36].

### Predictor variables

Sociodemographic variables like maternal characteristics (age, education, employment status), household characteristics (household wealth), spousal education and employment, sex of the child, antenatal care attendance of four or more times during the last pregnancy (ANC4 +), facility delivery and postnatal care practices (skin-to-skin contact immediately after delivery) were also included in the analysis based on evidence of their prior observed associations with breastfeeding practices [14, 37–39]. Wealth was measured using an asset-based index constructed from a principal components analysis using indicators measuring ownership of key consumer durables [40], and was rank-ordered and classified into quintile groupings.

### Data analysis

Descriptive analysis of the outcome with ideations and socio-demographic data were tabulated using survey weights to account for unequal probabilities of selection in the study sample.

We used mixed‐effects logistic regression models to separately quantify the association between the binary outcomes (early initiation of breastfeeding and EBF) with the main explanatory ideational variables (e.g. knowledge, beliefs, norms) adjusted for sociodemographic characteristics. All ideational and sociodemographic variables were included in the model as categorical fixed effects nested within a cluster identifier at the ward level. Prior to modelling, we tested for multicollinearity among variables using variance inflation factors. We used post-estimation analysis to calculate the predicted probabilities of early initiation of breastfeeding or EBF outcomes for respondents with or without certain ideations or sociodemographic characteristics [41]. The level of statistical significance was set to 0.05. STATA 16 (STATA Corporation, College Station, TX, USA) statistical software was used for all analysis.

## Results

### Study sample

A total of 3039 women with a completed pregnancy in the past 2 years resulting in a live birth responded to questions about their last-born child. Among the respondents, 42.1% (95% CI 35.1%, 49.4%) reported initiating breastfeeding within one hour of birth (early initiation of breastfeeding), and 37.5% (95% CI 29.8%, 46.0%) reported exclusive breastfeeding (EBF) of their last-born infant 0–5 months (*N* = 721). The mean age of women and their spouses were 26.0 years and 33.3 years respectively. Only 26.1% and 31.0% of women and their spouses had any formal education, with 50.3% and 91.9% working outside the home, respectively (Table 2).

**Table 2:** Study sample characteristics

	**Women with a child 0—5 months**	**Women with a child 6 – 23 months**	**Women with a child under 2 years**^a^
	%	%	%
	***N* = **721	*N* = 2277	*N* = 3039
**Respondent demographics**			
Age in years (mean)	25.7	26.1	26.0
Any formal schooling, primary attendance or higher	23.5	26.8	26.1
Employment outside home or student	48.2	51.0	50.3
Early initiation of breastfeeding	41.6	42.1	42.1
Exclusive breastfeeding	37.5	(…)	(…)
**Child demographics**			
Gender, female	45.1	48.5	47.8
**Spousal demographics**			
Age in years (mean)	33.1	33.5	33.3
Any formal schooling, primary attendance or higher	35.3	29.9	31.0
Employment outside home or student	93.5	91.3	91.9

^a^Includes women with a child from 0–24 months

(…), EBF calculated only for women with a child 0–5 months

### Descriptive analysis of early initiation of breastfeeding and EBF by ideational variables

Among women who knew that early initiation of breastfeeding protects the health of the newborn nearly half (47.7%) practiced early initiation of breastfeeding compared to 31.7% who did not know. In addition, 33.3% of women who believed that colostrum was bad milk practiced early initiation of breastfeeding compared to 45.3% who did not (Table 3).

**Table 3:** Descriptive analysis of early initiation of breastfeeding and EBF by Ideational variables

**Variable**	**Response categories**	**Women with children under 2 years (N)**	**Early Initiation of Breastfeeding % (95% CI)**^b^	**Women with children 0–5 months old (N)**	**EBF % (95% CI)**^b^
**Total**		**3039**	**42.1 (35.1, 49.4)**	**721**	**37.5 (29.8, 46.0)**
Spontaneously reports any benefits of EBF practice (first six months of infant’s life) for the mother	Any benefit	1494	46.4 (36.5, 56.6)	376	53.5 (43.3, 63.5)
	No benefits	1545	37.4 (29.6, 45.9)	345	18.3 (11.8, 27.4)
Spontaneously mentions immediate breastfeeding as a method to protect the health of the newborn after delivery	Yes	1919	47.7 (39.4, 56.3)	454	46.0 (37.0, 55.3)
	No	1120	31.7 (22.9, 42.0)	267	23.7 (15.1, 35.0)
Ideal age to introduce complementary foods in addition to breastmilk	6 Months	891	50.6 (39.0, 62.2)	228	55.4 (45.6, 64.8)
	Other responses	2148	38.3 (31.2, 45.9)	493	28.7 (20.2, 39.0)
Agreed (strongly or somewhat) that breastmilk contains all the nutrients a baby needs in first six months of life	Agree	2567	42.3 (35.0, 50.1)	592	43.2 (35.0, 52.0)
	Disagree	472	40.5 (26.9, 55.8)	129	9.9 (3.8, 23.8)
Agreed (strongly or somewhat) that mother's breastmilk after birth is bad milk	Agree	898	33.3 (24.3, 43.7)	206	25.5 (15.3, 39.3)
	Disagree	2141	45.3 (37.3, 53.5)	515	42.3 (34.4, 50.6)
Agreed (strongly or somewhat) on the importance of mothers to give their child only breastmilk in the first six months of infant’s life	Agree	1777	44.1 (35.4, 53.3)	432	50.5 (41.9, 59.1)
	Disagree	1262	38.6 (29.8, 48.3)	289	13.8 (8.6, 21.6)
Confident (very or somewhat) to practice EBF for first six months of infant’s life	Confident	1508	46.0 (37.0, 55.4)	388	52.4 (42.0, 62.7)
	Uncertain	1531	37.1 (29.4, 45.7)	333	16.2 (10.1, 25.1)
Confident (very or somewhat) to start conversation with partner about breastfeeding the child	Confident	2016	44.3 (36.4, 52.6)	493	46.0 (36.9, 55.3)
	Uncertain	1023	36.9 (27.5, 47.6)	227	15.3 (9.0, 25.0)
Agreed (strongly or somewhat) that most women in the community give infants only breastmilk in first six months of life	Agree	1193	48.2 (37.2, 59.3)	318	50.1 (39.8, 60.4)
	Disagree	1846	37.7 (30.5, 45.5)	403	27.0 (18.6, 37.6)
Does your partner influence your decision to breastfeed	Yes	1811	40.0 (31.6, 48.8)	458	41.8 (32.0, 52.3)
	No	1228	45.3 (35.7, 55.4)	263	30.1 (21.0, 41.3)
Does your mother influence your decision to breastfeed	Yes	230	53.8 (39.0, 67.8)	64	31.1 (16.4, 51.1)
	No	2809	41.2 (34.1, 48.7)	657	38.0 (29.8, 47.0)
Does your mother-in-law influence your decision to breastfeed	Yes	255	44.5 (35.0, 54.7)	68	30.1 (19.0, 44.0)
	No	2784	41.9 (34.7, 49.4)	653	38.1 (30.0, 47.1)
Does your healthcare provider influence your decision to breastfeed	Yes	135	61.0 (49.3, 71.5)	25	71.7 (49.4, 86.7)
	No	2904	41.0 (33.8, 48.6)	696	36.0 (28.1, 44.6)
**Socio-demographics**					
Household wealth	Lowest	716	39.0 (29.0, 50.0)	175	18.8(11.0, 30.2)
	Second	604	35.7 (27.3, 45.1)	143	36.6 (25.0, 50.0)
	Middle	600	31.9 (23.5, 41.6)	130	37.6 (23.7, 54.0)
	Fourth	496	53.0 (41.2, 63.6)	108	42.4 (30.3, 55.4)
	Highest	623	52.1 (43.5, 60.7)	165	51.0 (41.6, 60.4)
Maternal education^a^	Any formal education	499	53.9 (45.4, 62.2)	114	54.0 (43.6, 63.9)
	None or informal	2540	40.0 (32.6, 47.7)	607	34.5 (26.1, 44.0)
Maternal age (in years)	15–24 years	1275	37.5 (29.7, 46.0)	293	32.2 (21.7, 44.8)
	25- 34 years	1376	45.7 (38.3, 53.2)	341	40.8 (32.2, 49.9)
	35—49 years	388	44.2 (35.6, 53.1)	87	43.2 (29.1, 58.6)
Maternal occupation	Work outside home or student	1469	43.7 (34.4, 53.4)	337	37.0 (28.5, 46.4)
	No work outside home	1409	40.4 (32.0, 49.5)	345	40.9 (29.1, 54.0)
	Other	111	50.7 (20.8, 80.0)	29	28.5 (6.8, 68.5)
	Missing	50		10	
Child sex (gender)	Male	1579	42.0 (34.6, 50.0)	377	37.9 (29.2, 47.5)
	Female	1460	42.1 (35.0, 49.6)	344	37.0 (29.0, 46.0)
Spousal occupation	Work outside home or student	2742	41.9 (35.0, 49.2)	650	38.4 (30.8, 46.7)
	No work outside home	149	42.1 (25.8, 60.3)	38	44.7 (28.3, 62.3)
	Other	98	57.0 (42.0, 70.8)	23	29.1 (8.6, 64.1)
	Missing	50		10	
Spousal education^a^	Any formal education	933	48.0 (39.7, 56.4)	243	49.1 (39.4, 59.0)
	None or informal	2056	40.0 (32.4, 48.1)	468	32.5 (23.7, 42.7)
	Missing	50		10	
Facility delivery	Yes	520	48.2 (38.7, 57.7)	139	47.5 (35.7, 59.5)
	No	2507	41.1 (33.6, 49.0)	577	35.7 (27.4, 45.1)
	Missing	12		5	
Skin-to-skin contact after delivery	Yes	905	45.3 (34.3, 56.9)	212	40.1 (30.5, 50.4)
	No	2134	41.0 (33.1, 49.3)	509	36.7 (27.8, 46.6)
ANC4 + attendance	Yes	712	55.4 (47.4, 63.2)	174	53.4 (41.9, 64.7)
	No	2313	37.9 (30.3, 46.1)	544	32.0 (23.2, 42.4)
	Missing	14		3	

*EBF* Exclusive Breastfeeding, *ANC4* + Antenatal Care Attendance of four or more times, *CI* Confidence Interval

^a^Formal education includes primary, secondary or tertiary education; none/informal education includes Islamic education or no formal education

^b^Row percentage

Regarding ideations for EBF, 46.0% of women who knew that early initiation of breastfeeding protects the health of the newborn practiced EBF compared to 23.7% who did not know. Furthermore, among the women who believed that colostrum was bad milk, one-quarter (25.5%) practiced EBF compared to 42.3% who did not, while nearly three-quarters (71.7%) of women who cited health providers as a social influence on their breastfeeding decisions practiced EBF compared to 36.0% who did not (Table 3).

### Reasons for not practicing EBF

**Fig. 1:**
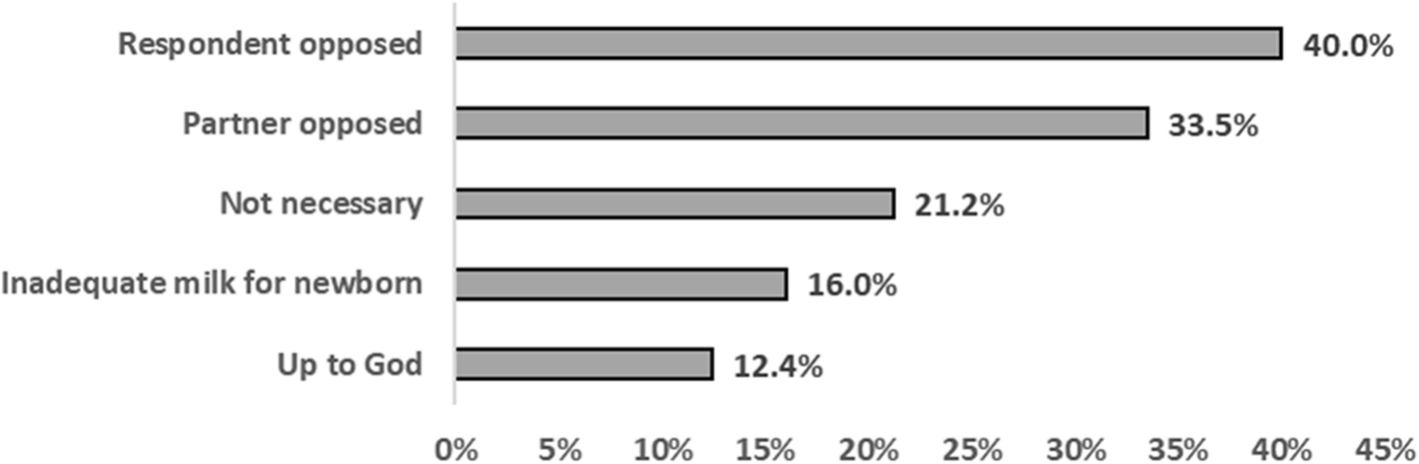
Reasons for not practicing EBF for the first six months of infant’s life

We also asked women who did not exclusively breastfeed their child, reasons for not doing so. Among these women (Fig. 1), the most commonly cited reasons were: personal opposition to EBF (40.0%, 95% CI 31.3%, 49.4%), spousal disapproval of EBF (33.5%, 95% CI 26.2%, 42.0%), respondents not perceiving EBF as necessary (21.2%, 95% CI 15.0%, 27.6%), and also respondents’ perception of breastmilk alone as inadequate milk for newborn (16.0%, 95% CI 10.2%, 23.7%). We did not further explore these constraints to the practice of EBF.

### Association between ideational factors and early initiation of breastfeeding

Based on regression analyses, the significant ideations associated with the early initiation of breastfeeding outcome were maternal knowledge and beliefs about the health benefits of early initiation of breastfeeding and also mothers’ beliefs about colostrum (Table 4, left panel). After adjustments for other factors, women who knew at least one benefit of EBF to themselves had a 5.1 percentage point (pp) higher probability of practicing early initiation of breastfeeding (95% CI 0.8%, 9.4%) compared to those who did not. Furthermore, women who knew that early initiation of breastfeeding protects the health of the newborn had a 7.9 pp higher probability of practicing early initiation of breastfeeding (95% CI 3.9%, 11.9%) compared to those that did not know. In contrast, women who believed that colostrum was bad milk had an 8.4 pp lower likelihood (95% CI -12.4%, -4.3%) of practicing early initiation of breastfeeding compared to those who believed otherwise. With respect to socio-demographic characteristics, maternal employment and ANC4 + attendance were positively associated with early initiation of breastfeeding in the adjusted analysis.

**Table 4:** Ideational and sociodemographic associations with early initiation of breastfeeding and EBF practice

		**Early Initiation of Breastfeeding**				**EBF**			
**Variable**	**Response categories**	**Average marginal effect**	**95% CI**		***P*-value**	**Average marginal effect**	**95% CI**		***P*-value**
**Maternal ideations**									
Spontaneously reports any benefits of EBF practice (first six months of infant’s life) for the mother	Any benefit	0.051	0.008, 0.094		**0.021**	0.133	0.050, 0.220		**0.003**
	No benefit	-							
Spontaneously mentions immediate breastfeeding as a method to protect the health of the newborn after delivery	Yes	0.079	0.039, 0.119		** < 0.001**	0.070	-0.011, 0.144		0.094
	No	-				-			
Ideal age to introduce complementary foods in addition to breastmilk	6 Months	0.030	-0.008, 0.067		0.127	0.112	0.039, 0.185		**0.003**
	Other responses								
Agreed (strongly or somewhat) that breastmilk contains all the nutrients a baby needs in first six months of life	Agree	0.037	-0.015, 0.090		0.165	0.047	-0.065, 0.160		0.409
	Disagree	-				-			
Agreed (strongly or somewhat) that mother's breastmilk after birth is bad milk	Agree	-0.084	-0.124, -0.043		** < 0.001**	-0.084	-0.157, -0.010		**0.026**
	Disagree								
Agreed (strongly or somewhat) on the importance of mothers to give their child only breastmilk in the first six months of infant’s life	Agree	-0.016	-0.060, 0.027		0.458	0.071	-0.019, 0.160		0.124
	Disagree	-				-			
Confident (very or somewhat) to practice EBF for first six months of infant’s life	Confident	-0.002	-0.050, 0.045		0.920	0.131	0.032, 0.230		**0.010**
	Uncertain								
Confident (very or somewhat) to start conversation with partner about breastfeeding the child	Confident	0.016	-0.030, 0.062		0.478	0.021	-0.077, 0.119		0.673
	Uncertain	-				-			
Agreed (strongly or somewhat) that most women in the community give infants only breastmilk in first six months of life	Agree	-0.011	-0.053, 0.031		0.610	0.004	-0.073, 0.081		0.920
	Disagree								
Does your partner influence your decision to breastfeed	Yes	-0.010	-0.050, 0.029		0.599	-0.020	-0.098, 0.058		0.611
	No	**-**				**-**			
Does your mother-in-law influence your decision to breastfeed	Yes	-0.053	-0.107, 0.002		0.060	-0.028	-0.132, 0.076		0.594
	No	**-**				**-**			
Does your mother influence your decision to breastfeed	Yes	0.040	-0.023, 0.101		0.217	-0.028	-0.138, 0.083		0.623
	No	**-**				**-**			
Does your healthcare provider influence your decision to breastfeed	Yes	0.026	-0.052, 0.104		0.513	0.237	0.033, 0.440		**0.023**
	No	**-**				**-**			
**Socio-demographics**									
Household wealth	Lowest	-				-			
	Second	-0.018	-0.064, 0.029		0.455	0.003	-0.093, 0.099		0.948
	Middle	-0.027	-0.080, 0.025		0.313	0.035	-0.072, 0.142		0.522
	Fourth	0.048	-0.011, 0.106		0.110	0.044	-0.107, 0.116		0.938
	Highest	0.018	-0.047, 0.082		0.594	0.002	-0.117, 0.120		0.979
Maternal education^a^	Any formal education	0.028	-0.023, 0.080		0.283	-0.020	-0.113,0.072		0.666
	None or informal	-				-			
Maternal age (in years)	15–24 years	-				-			
	25—34 years	0.027	-0.005, 0.058		0.098	0.023	-0.044, 0.090		0.499
	35—49 years	0.031	-0.016, 0.080		0.283	-0.058	-0.151, 0.035		0.224
Maternal occupation	Work outside home or student	0.052	0.006, 0.098		**0.027**	0.018	-0.058, 0.094		0.641
	No work outside home	-				-			
	Other	0.023	-0.084, 0.130		0.674	0.018	-0.167, 0.203		0.849
Child sex (gender)	Male	0.005	-0.023, 0.035		0.689	-0.006	-0.064, 0.052		0.828
	Female	-				-			
Spousal occupation	Work outside home or student	0.025	-0.047, 0.097		0.500	-0.052	-0.193, 0.089		0.472
	No work outside home	-				-			
	Other	0.062	-0.053, 0.178		0.291	-0.166	-0.374, 0.043		0.119
Spousal education^a^	Any formal education	-0.035	-0.077, 0.007		0.100	0.055	-0.028, 0.137		0.193
	None or informal	-				-			
Facility delivery	Yes	-0.033	-0.080, 0.014		0.172	-0.020	-0.103, 0.063		0.638
	No	-				-			
Skin-to-skin contact after delivery	Yes	0.022	-0.020, 0.064		0.313	-0.039	-0.115, 0.037		0.315
	No	-				-			
ANC 4 +	Yes	0.066	0.024, 0.109		**0.002**	0.038	-0.041, 0.117		0.346
	No	-				-			

Significant results in bold

*EBF* Exclusive Breastfeeding, *ANC4* + Antenatal Care Attendance of four or more times, *CI* Confidence Interval

^a^Formal education includes primary, secondary or tertiary education; none/informal education includes Islamic education or no formal education

(-) denotes the reference category

### Association between ideational factors and EBF

The significant ideational variables associated with EBF for the first six months of life were knowledge about breastfeeding benefits to the mother and newborn, knowledge about the ideal age to introduce complementary foods; self-efficacy to practice EBF for the first six months of life; beliefs about colostrum as bad milk and influence of health providers on breastfeeding decisions (Table 4, right panel).

In terms of the cognitive domain, the probability of practicing EBF was 13.3 pp higher (95% CI 5.0%, 22.0%) among women who knew at least one benefit of EBF to themselves compared to those who did not. Also, women who knew that six months was the ideal age to introduce complementary feeding had a 11.2 pp higher probability of practicing EBF (95% CI 3.9%, 18.5%) compared to those who gave a different response. Finally, women who believed that colostrum was bad milk had a 8.4 pp lower likelihood (95% CI -15.7%, -1.0%) of practicing EBF compared to those who believed otherwise.

Regarding emotional and social domains, women who felt confident to practice EBF were 13.1 pp (95% CI 3.2%, 23.0%) more likely to practice EBF for the first 6 months of life than those who were uncertain. Lastly, women who reported that health providers mainly influenced their decision to breastfeed had a 23.7 pp increase (95% CI 3.3%, 44.0%) in the probability to practice EBF compared to those who did not report health worker influence.

## Discussion

Since breastfeeding is a cultural norm in northwestern Nigeria [42], and the benefits of breastfeeding to both mother and infant are well known within our study region [43], the existing challenge is to ‘shift’ the current early initiation of breastfeeding and EBF practices closer to optimal breastfeeding levels. This means emphasizing the benefits of timely initiation of breastfeeding after delivery and delaying the introduction of any liquids (including water) and foods, for six months. Overall, we found low rates of early initiation of breastfeeding and EBF practices in our study area among women 15–49 years during their most recent pregnancy in the last two years.

Consistent with the Ideation Model of Strategic Communication and Behavior Change [15], the various cognitive, emotional and social domains played a crucial role in women’s breastfeeding decisions. Specifically, we found that maternal knowledge about the benefits of breastfeeding to the mother (cognitive), knowledge of the appropriate time to introduce complementary foods (cognitive), beliefs on colostrum (cognitive), self-efficacy to breastfeed (emotional) and perceived social norms (social) are among the most important ideations for SBC programs to target to increase early initiation of breastfeeding and EBF rates in northwestern Nigeria.

In the cognitive domain, we found that a mother’s knowledge of the benefits of EBF to herself (knowledge), knowledge of the protective effect of immediate breastfeeding on the health of her newborn (knowledge) and beliefs around colostrum (beliefs) had significant effects on the mother’s decision to practice both early initiation of breastfeeding and EBF. Previous studies from northwestern Nigeria have linked high maternal knowledge of the benefits of EBF with a high prevalence of practicing early initiation of breastfeeding and EBF [44, 45]. Negative beliefs around colostrum was a risk factor for early initiation of breastfeeding and EBF in our study as the widespread belief that colostrum was bad milk led to the delay in early initiation of breastfeeding an also lower likelihood to practice EBF. It is well documented that women in the northwestern Nigeria region may consider colostrum harmful to the infant and therefore delay initiation of breastfeeding [46]. The study by Patel et al. [47] enhanced acceptability of colostrum by improving maternal breastfeeding education. In Southern Asia, engaging with social and family decision-makers to change perceptions around colostrum was also recommended [48]. Therefore, SBC programs would need to move beyond the emphasis on the benefits of breastfeeding (knowledge) to address cognitive beliefs that may impede progress in improving early initiation of breastfeeding and EBF rates within this region, such as dispelling misperceptions about colostrum as bad milk and also engaging community leaders and family decision makers to shape beliefs and attitudes towards safer and optimal breastfeeding practices.

Furthermore, along the cognitive domain, knowledge of the appropriate time to introduce complementary foods (at six months of age) was a driver of the decision to practice EBF but not early initiation of breastfeeding. It has been well documented in Nigeria that optimal exclusive breastfeeding practices were positively associated with timely initiation of breastfeeding practices [49]. Studies from other settings [50, 51] have shown that postnatal maternity care which involves counselling on child care, breastfeeding and infant feeding practices was associated with improved knowledge of the appropriate time to introduce complementary feeding and timely initiation of complementary feeding.

Within the social domain, the belief that EBF is an important practice (injunctive norms) was also significantly associated with EBF practice but not early initiation of breastfeeding. Our findings are consistent with previous studies [52, 53] which reported that Infant and Young Child Feeding (IYCF) information diffused through social networks, led to positive changes in the practice of EBF. Our findings present an opportunity for SBC interventions to promote and sustain messaging on positive breastfeeding behaviors in the communities, leveraging on the mother’s social networks.

It has also been well documented that when healthcare personnel provide encouragement and support on breastfeeding to women, the likelihood of initiating and sustaining breastfeeding increases [54, 55]. Health providers’ supportive influence was positively associated with EBF practice but not early initiation of breastfeeding. This may partly be explained by the fact that approximately 84% of the women in our study delivered outside the health facility and the implication of this being that their deliveries may not have been assisted by a health personnel. However, facility delivery was not a significant predictor of either early initiation of breastfeeding or EBF practice in our study.

The 2018 NDHS [5] reported a higher prevalence of early initiation of breastfeeding (50%) among children delivered in the presence of a skilled birth attendant/health personnel compared to those delivered in the presence of a traditional birth attendant (33%), no one (36%) or others (37%). Our findings are also consistent with previous reviews of randomized controlled trials [56] that showed professional-led support from health providers had a greater impact on the practice of EBF compared to peer-led support. SBC programs need to promote the importance of utilizing health facilities for delivery in the communities and also expand breastfeeding promotion through health providers by specifically targeting women during ANC counseling sessions [55].

Regarding the emotional domain, maternal self-efficacy to practice EBF was significantly associated with EBF practice but not early initiation of breastfeeding. The literature [57, 58] has consistently shown that a woman’s level of breastfeeding self-efficacy is strongly associated with EBF duration*.* SBC interventions should focus on improving mother’s self-efficacy to exclusively breastfeed by including self-efficacy interventions as part of their programs.

The difference in our findings between ideations related to the practice of early initiation of breastfeeding and EBF may be partly explained by the widespread belief by some women that colostrum is bad milk. This singular factor may have negatively influenced their decision to practice early initiation of breastfeeding despite knowing the importance of breastfeeding and having self-efficacy to practice EBF.

### Study limitations

Our study has some limitations that warrant mentioning. Firstly, our findings are based on cross-sectional data and therefore should be interpreted with caution with regards to making a causal influence of the ideational determinants. It is possible that our outcomes (early initiation of breastfeeding or EBF practice) may have informed the ideational determinants. Secondly, as with cross-sectional surveys, there could be residual confounding such that a confounding variable was unmeasured or incorrectly measured such that making adjustments for that variable in the model is difficult. This could potentially introduce bias into the estimates of the marginal effects of our regression models’ covariates. Lastly, responses to ideational questions may be affected by social desirability bias, respondents’ moods or recent experiences at the time of the interview.

### Conclusion and recommendations

Improving women’s knowledge about breastfeeding and its benefits to themselves and their infants (knowledge), improving maternal education on the appropriate timing to introduce complementary feeding to their infants (Knowledge), dispelling myths around colostrum as bad milk (beliefs), building women’s confidence to exclusively breastfeed (self-efficacy), and leveraging on the influence of health providers (social influence) are the most important ideations for SBC programs to target to improve early initiation of breastfeeding and EBF breastfeeding behaviors for women in northwestern Nigeria.

Indeed, ideations like knowledge (cognitive), beliefs (cognitive), self-efficacy (emotional) and social influence (social) under the cognitive, emotional and social ideational dimensions provide significant insights for SBC programs and policy makers to improve health behaviors around breastfeeding practices. Ideational metrics should be considered for examination in other health areas.

## Data Availability

The data that supports the findings of the current study are available from the corresponding author upon reasonable request.

## Abbreviations

ANC: Antenatal Care
ANC4 +: Antenatal care attendance at least four or more times during pregnancy
EBF: Exclusive Breastfeeding
EIBF: Early Initiation of Breastfeeding
IYCF: Infant and Young Child Feeding
MNCH + N: Maternal,Newborn & Child Health + Nutrition
NDHS: Nigeria Demographic and Health Survey
SBC: Social and Behavior Change
SBCC: Social and Behavior Change Communication
