# Correction: The role of maternal ideations on breastfeeding practices in northwestern Nigeria: a cross-section study

**DOI:** 10.1186/s13006-022-00512-6

**Published:** 2022-11-04

**Authors:** Udochisom C. Anaba, Emily White Johansson, Dele Abegunde, Gloria Adoyi, Olayinka Umar-Farouk, Shittu Abdu-Aguye, Paul C. Hewett, Paul L. Hutchinson

**Affiliations:** 1 Department of Global Community Health and Behavioral Sciences, School of Public Health and Tropical Medicine, Tulane University, New Orleans, USA; 2 Breakthrough RESEARCH/Nigeria, Plot 839 Idris Ibrahim Crescent, Abuja, Nigeria; 3 Population Council, Washington DC, USA; 4 Breakthrough ACTION/Nigeria, Abuja, Nigeria; 5 Save the Children, Abuja, Nigeria; 6 Johns Hopkins Center for Communication Programs, Baltimore, USA; 7 Formerly Population Council, Washington DC, USA

International Breastfeeding Journal (2022) 17:63 10.1186/s13006-022-00500-w

Following publication of the original article [1], the authors flagged that ‘Formerly’ had been erroneously included in affiliation 3. The published article has since been corrected and the corrected affiliation may be seen in this erratum.

